# Hospital productivity bias when not adjusting for cost heterogeneity: The case of Spain

**DOI:** 10.1371/journal.pone.0218367

**Published:** 2019-06-18

**Authors:** Alejandro Arvelo-Martín, Juan José Díaz-Hernández, Ignacio Abásolo-Alessón

**Affiliations:** 1 Grupo de Investigación de Economía Pública y de la Salud, Universidad de La Laguna, Santa Cruz de Tenerife, España; 2 Departamento de Economía, Contabilidad y Finanzas, Instituto Universitario de Desarrollo Regional, Universidad de La Laguna, Santa Cruz de Tenerife, España; 3 Departamento de Economía Aplicada y Métodos Cuantitativos, Instituto Universitario de Desarrollo Regional, Campus de Guajara, Tenerife, Spain; Institute for Economic Forecasting, Romanian Academy, ROMANIA

## Abstract

This research quantifies the bias caused in hospital productivity measurements when cost heterogeneity is not considered. A multi-output stochastic cost frontier under a normalised translog specification is used to approximate the structure of technology of a sample of public general hospitals in Spain during the period 2002–2009. To control for observable heterogeneity in costs, a set of variables related to hospital characteristics are included in the cost frontier specification (i.e., hospital complexity, degree of specialisation, availability of outpatient clinics, variety of high-technology equipment available, teaching activity and quality of care), whereas unobservable heterogeneity is accounted for by means of individual dummy variables. A measure of hospitals’ cost efficiency is first obtained, and the analysis is then completed by measuring and decomposing the total factor productivity index (TFP-I) change. Findings reveal that controlling for heterogeneity decreases total productivity from an annual average rate of 0.028% to 1.330%, mainly driven by the negative contribution of the cost efficiency change component. Hence, a bias of 1.303 percentage points in the overall TFP-I is found as consequence of not controlling for heterogeneity. In addition to this, if heterogeneity factors are not accounted for, the mean cost efficiency index during the period analysed is 0.730, figure that increases up to 0.974 if heterogeneity is considered. Hence, the omission of heterogeneity leads to a bias of 24.4 percentage points in the mean cost efficiency. Therefore, not adjusting for heterogeneity in costs gives rise to distorted measurements of hospital productivity, as well as distortions in the contribution of each of its components, which may lead to the adoption of inadequate policies and decisions on resource allocation.

## Introduction

In the literature on hospital performance, increasing attention has been paid to the fact that hospital production activity could be influenced by factors other than merely outputs and inputs. Thus, hospitals’ characteristics and elements inherent to the environment where units operate in–e.g., hospital complexity, teaching status, degree of specialization, etc.—may affect their costs and performance [1]. As performance measures can be regarded as success indicators [2], accounting for the effects of these hospital cost heterogeneity factors turns out to be crucial to avoid misleading conclusions that may affect policies designed to improve hospital productive processes.

Hospital performance has been commonly approached through productivity measurements. Indeed, the need to obtain productivity measurements in a multiple-input and multiple-output context, such as that of hospitals, has given rise to the concept of Total Factor Productivity (TFP) index. This index measures the performance of a unit by the quotient of an aggregated measure of the outputs produced relative to an aggregated measure of the inputs used. Moreover, the TFP index accounts for other factors that may influence productivity, such as the scale in which units operate, i.e., economies of scale—or investments in technology and organizational adjustments, i.e., technical change. Therefore, the TFP index provides more comprehensive information on both hospitals’ cost structure and performance than just efficiency measurements [3].

In addition to efficiency, *frontier* methods can provide productivity measurements. Thus, TFP indexes can also be derived by obtaining a frontier of reference using either *non-parametric* or *parametric* techniques [4]. Although approaches using parametric methods and measurements and decomposition of productivity have been developed in the literature on performance measurements [5–6], the majority of applications to hospital productivity have employed non-parametric methods to obtain the frontier, and thus have addressed the construction of a productivity index based on the Malmquist Index [7–10].

Hospital cost heterogeneity has been extensively considered in frontier analyses on hospital performance. However, although there is little evidence of measurement bias caused in hospital efficiency indexes by the omission of these factors, we are not aware of any previous study measuring the bias in hospital productivity levels. The premise is that hospitals operating in relatively more adverse conditions will be located at a greater distance from the benchmark frontier, appearing to be more inefficient. In other words, not accounting for elements of heterogeneity in costs is likely to give rise to higher inefficiency values [11] and, in turn, to lower productivity values. In addition, heterogeneity factors could also affect the use of technology and scale of operations. Thus, these elements may also influence hospital productivity via their impact on its components of technical change and scale economies.

Considering all the above, the main objective of this research is to estimate -in the context of Spanish public general hospitals- the bias that the omission of hospital cost heterogeneity may cause in measurements of hospital productivity as well as in each of its components. The Spanish public health system is a national health system (SNHS) characterised by universal coverage and tax funding. In addition, the SNHS is decentralised in such a way that all 17 Spanish regions (Autonomous Communities) have the responsibility to manage, regulate and plan the provision of health care services for their respective populations, leaving basically the coordination tasks to the central government. This decentralisation took more than 20 years: from 1981, when Cataluña took over the management of its health care system, to 2002, when the health services were devolved to the last ten Autonomous Communities depending on the central government. Based on hospital data from the SNHS, a stochastic cost frontier is estimated following parametric techniques to derive measures of hospital cost efficiency and productivity. This constitutes an adequate methodology for the purposes of this study as: (1) it enables to test hypotheses on the existence of hospital cost heterogeneity and (2) it allows the separation of effects related to random shocks from inefficiency, and by extension, from productivity measures.

The remainder of the paper is organised as follows. Section 2 provides a review of how previous literature on hospital performance has accounted for the effects of hospital cost heterogeneity. In section 3, the methodology followed, as well as the description of the variables used, are outlined. Results of the analysis are reported in section 4, whereas these are discussed in section 5 that includes the concluding remarks.

## Background

The effects of hospital cost heterogeneity have been extensively controlled for in previous literature on hospital performance following a stochastic frontier approach. In particular, two types of hospital cost heterogeneity can be distinguished: on the one hand, differences in costs whose causative factors–other than input and outputs- can be identified (i.e., observable heterogeneity). These elements have been commonly captured by including them as a vector of exogenous variables in the model chosen to analyse the optimal productive behaviour. The list of variables frequently used to control for these elements is broad. For example, Street et al. [1] have cited, as possible determinants of hospital costs, factors related to hospital characteristics, such as teaching status and quality of care. On the other hand, differences in hospital costs arising from elements that cannot be associated with any possible known factor (i.e., unobserved heterogeneity). This latter type of heterogeneity has been traditionally controlled for by means of individual dummy variables.

Previous literature has also accounted for the effects of hospital complexity (for example, see [12–17]) and the degree of specialisation (for example, see [18–19]). The idea that hospitals attending more complex cases are likely to present higher costs is widely accepted, with a measure of the hospital case-mix being an extensively used proxy to control for hospital complexity. Thus, higher costs for hospitals attending more complex cases have been reported in Spain [12–13,15] and in U.S. [16–17,20–23].

The degree of specialisation is also an issue of increasing interest in hospital performance literature. Hospitals specialised in the provision of a particular health care service would benefit from the expertise of staff undertaking the same procedures over time. Also, cost savings may derive from the absence of competition for the use of resources [1]. Thus, Daidone and D’Amico [18], who computed a specialisation index in the Major Diagnosis Category (MDC) based on the Gini Index, found a decreasing impact of specialisation on hospital costs in a sample of Italian hospitals. However, Vitikainen, Linna and Street [19], using the share of the largest MDC in terms of costs of overall production as proxy for the degree of specialisation, found no significant influence on hospital costs.

The possibility of providing care in a regime that does not require in-hospital stays, which can be regarded as an important source for cost containment, may have significant effects on hospital performance. However, García [24], using a binary choice variable, found an increasing effect on hospital costs derived from the availability of outpatient clinics associated with hospitals in Spain.

The availability of technology equipment can be regarded as a measure of a hospital’s capacity to respond to demand. In this respect, the variety of high-technology equipment for the diagnosis and treatment could lead to higher efficiency levels [25]. In this regard, González et al. [26] accounted for the effects of the availability of high-technology equipment on hospital costs in Spain.

Teaching status has also been extensively considered to significantly influence hospital performance. The generally accepted premise is that those hospitals providing education and training for doctors and other healthcare personnel will obviously face additional costs, such as investments in material, equipment and facilities for teaching purposes [27]. This feature has been traditionally controlled for by including a dummy variable or the number of resident doctors. When accounting for the effect on hospital technology of production, higher levels of costs in hospitals with teaching activity have been found in Spain [13,15,28], in U.S. [17, 20–22] and in Finland [19].

There is a generalised idea on the existence of a trade-off between quality of care and efficiency. As personnel devoted to care provision has been used as a quality measure in the literature on hospital performance, those hospitals providing care with a relatively higher number of personnel may pursue the provision of more costly hospital care but also higher-quality care. In this regard, Farsi and Filippini [29] and Daidone and D’Amico [18] showed that higher values of the number of nurses per bed have decreased efficiency levels in primary and hospital care, respectively.

In the related literature, it has recently been stressed that, in addition to observable sources of cost heterogeneity, unobserved factors may also have a significant effect on levels of hospitals’ performance. Widmer, Zweifel and Farsi [30] found an increase of six percentage points, on average, in cost efficiency indexes in a sample of Swiss hospitals during the period 2004–2007 when unobservable heterogeneity was accounted for. Subsequently, Widmer [31] found that accounting for unobserved factors raised cost efficiency indexes by two percentage points, on average, in a sample of Swiss hospitals during the period 2004–2009.

Studies based on hospital productivity have also focused on measuring and decomposing productivity. Thus, applications of this approach on hospital productivity in U.K., Sweden, U.S., Taiwan and South Africa can be identified in the reviews of Hollingsworth, Dawson and Maniadakis [7] and Hollingsworth [8,10]. However, we have not been able to identify any previous application explicitly addressing the measurement of bias introduced in productivity indexes as a consequence of the omission of heterogeneity in hospital costs. Moreover, in the Spanish context, none of the studies estimating a stochastic cost frontier for hospital performance measurement has calculated and decomposed productivity change. Rather, the existing literature is mostly based on the Malmquist Index alongside DEA methodology for inefficiency modelling.

## Method, data and variables

### Method

A parametric technique is followed in this research, and the variation of the TFP index change is calculated and decomposed into cost efficiency change, technical change and scale economies effect following the approach proposed by Bauer [6], so that:

$$\overset{\circ }{TFP}=\left[1-\sum_{m}\varepsilon_{cy_{m}}(y,w,t)\right]\overset{\circ }{y^{c}}+\overset{\circ }{CE}-\overset{\circ }{c}(y,w,t)+\sum_{n}\left[s_{n}-s_{n}(y,w,t)\right]\overset{\circ }{w_{n}}+\left(\overset{\circ }{y^{p}}-\overset{\circ }{y^{c}}\right)$$ (1)

The first component, $\left[1-\sum_{m}\varepsilon_{cy_{m}}(y,w,t)\right]\overset{\circ }{y^{c}}$ provides a measure of the effects of scale economies on productivity change, where $\sum_{m}\varepsilon_{cy_{m}}(y,w,t)=\sum_{m}\left[\frac{\partial LnTC_{it}}{\partial Lny_{mit}}\right]$ and $\overset{\circ }{y^{c}}=\sum_{m}\left[\frac{\varepsilon_{cy_{m}}}{\sum_{m}\varepsilon_{cy_{m}}}\right]\frac{\partial y_{m}}{\partial t}$. Notice that scale economies $\sum_{m}\varepsilon_{cy_{m}}(y,w,t)<1$ lead to increases in productivity via increases in output levels. The second component measures the contribution of cost efficiency changes to productivity growth, and it is obtained as $C\overset{\circ }{E}=\frac{\partial CE}{\partial t}$. The third component, $\overset{\circ }{c}(y,w,t)$, captures the variations in productivity levels due to technical change. This component is obtained as $\overset{\circ }{c}=\left(\frac{\partial c}{\partial t}\right)\frac{1}{c}=\varepsilon_{ct}(y,w,t)\frac{1}{t}$, where $\varepsilon_{ct}(y,w,t)=\frac{\partial LnTC_{it}}{\partial Lnt}$. Changes in each component are obtained as percentage variations. Finally, the last two terms in Eq (1) are residual terms. $\sum_{n}\left[s_{n}-s_{n}(y,w,t)\right]\overset{\circ }{w_{n}}$ provides a measure of the bias introduced by the aggregation of inputs using the observed input shares in place of efficient input shares. Moreover, since an aggregation of outputs is also required, an additional residual term capturing the bias introduced by the aggregation of outputs using the cost elasticity weights in place of revenue share weights, i.e., $\left(\overset{\circ }{y^{p}}-\overset{\circ }{y^{c}}\right)$, is included.

As the approach proposed by Bauer [6] requires the estimation of a frontier of reference against which to measure productive behaviour, hospitals’ structure of technology is approximated by means of the following multi-output stochastic long-run cost frontier under a normalised translog specification:

$$\begin{array}{l} Ln\left(TC_{it}(y,w,t)/w_{sit}\right)=\beta_{0}+\sum_{m}\beta_{m}Ln\;y_{mit}+\sum_{n\neq s}\beta_{n}Ln\left(w_{nit}/w_{sit}\right)+\\ \frac{1}{2}\sum_{m}\sum_{j}\beta_{mj}Ln\;y_{mit}Ln\;y_{jit}+\frac{1}{2}\sum_{n\neq s}\sum_{k\neq s}\beta_{nk}Ln\left(w_{nit}/w_{sit}\right)Ln\left(w_{kit}/w_{sit}\right)+\\ \sum_{m}\sum_{n\neq s}\beta_{mn}Ln\;y_{mit}Ln\left(w_{nit}/w_{sit}\right)+\beta_{t}Ln\;t+\frac{1}{2}\beta_{tt}Ln\;t^{2}+\sum_{m}\beta_{mt}Ln\;y_{mit}Ln\;t+\\ \sum_{n\neq s}\beta_{nt}Ln\left(w_{nit}/w_{sit}\right)Ln\;t+\sum_{p}\theta_{p}h_{pit}+\sum_{q}\rho_{q}Dhosp_{qi}+\nu_{it}+u_{it} \end{array}$$ (2)

Where *TC*_*it*_ is the observed total cost of hospital *i* in period *t y*_*mit*_, is the level of output *m* (i.e., inpatient, outpatient and emergency outputs), *w*_*nit*_ is the price of the input *n* (i.e., capital, labour, pharmacy and materials and services inputs) and *t* indicates systematic variations in total costs due to time (i.e., technical change). In order to control for differences in costs due to observable hospital characteristics, a vector of exogenous variables thought to affect hospital costs, *h*_*pit*_, is included in the deterministic part of the cost frontier (i.e., complexity; degree of specialisation; availability of an associated outpatient clinic; the variety of high-technology equipment available; teaching activity and quality of care). In addition to this, to control for those unobservable factors potentially affecting hospital costs, a set of individual dummy variables, *Dhosp*_*qi*_, has been also included in (2). Hence, we focus on the effects of both observable and unobservable cost heterogeneity characteristics and environmental factors on hospital technology of production rather than on inefficiency per se. The last two terms, *v*_*it*_ and *u*_*it*_, represent the statistical random noise and inefficiency, respectively. Model choice is made due to the flexibility of the translog specification, as it does not require any prior assumption on hospital technology characteristics and these can be tested after the estimation.

The specified cost frontier is estimated by previously ensuring that it satisfies the property of linear homogeneity in input prices. This is accomplished by normalising the cost and input prices, i.e., by dividing total cost and input prices by one input price, *w*_*sit*_. Constraints of symmetry are additionally imposed (i.e; *β*_*mj*_ = *β*_*jm*_ *β*_*nk*_ = *β*_*kn*_; *β*_*mn*_ = *β*_*nm*_; and *β*_*nt*_ = *β*_*tn*_). Moreover, cost, input prices and outputs are expressed in logarithms and deviation with respect to the sample mean. Consequently, first order parameters can be interpreted as cost elasticities for the sample mean hospital.

Cost frontier specified is estimated by Maximum Likelihood Estimation following the Battese and Coelli (1992) approach [32], which assumes that both error components are distributed, independently from each other and from the rest of covariates included in the model, as $u_{it}∼N(\mu ,\sigma_{u}^{2})$ and $\nu_{it}∼N(0,\sigma_{\nu }^{2})$. Moreover, this model allows for efficiency variation over time, such that *u*_*it*_ = *β*(*t*)*u*_*i*_, where *β*(*t*) = {exp[−*η*(*t*−*T*)]}, which implies that inefficiency decreases, increases or remains constant if η > 0, η < 0 and η = 0, respectively. Incorporating exogenous variables to control for heterogeneity into the cost frontier under a Battese and Coelli (1992) model follows the approach adopted by Linna (1998). By including an individual dummy variable, a fixed-effects version of the model is estimated here. Thus, a similar procedure as that used by Filippini and Wetzel [33], who estimated a fixed-effects version but using the Battese and Coelli (1995) model [34], is followed. Given the estimation problems of alternatives for accounting for unobserved heterogeneity, such as those of True-Fixed and True-Random Effects models proposed by Greene [35–36], which are based on Simulated Maximum Likelihood, the approach used here fulfils the methodological requirements for our research purposes.

In order to obtain a measure of the bias that may be introduced in the estimation process as a consequence of the omission of cost heterogeneity variables, two versions of the translog frontier specified in (2) are estimated: a restricted version (Model I) accounting only for outputs and input prices and an extended version (Model II) accounting for cost heterogeneity (i.e., both variables sets *h*_*pit*_ and *Dhosp*_*qi*_). We will test hypotheses on the convenience of controlling for sources of heterogeneity in costs to choose between the two models specified.

Estimates of *u*_*it*_ are derived as the mean of the conditional distribution of *u*_*it*_ given *ε*_*i*_, as proposed by Jondrow et al. [37]. Individual estimates of cost efficiency are then obtained as *CE*_*it*_ = exp(*u*_*it*_). Variation of *CE*_*it*_ over time will provide us with the contribution of cost efficiency change to hospital productivity change. Economies of scale effects and technical change are calculated as indicated in Eq (1) using cost frontier estimates. The cost frontier as well as the individual inefficiency measures are estimated by means of the stochastic frontier estimation routines provided by STATA 13 (for more details, see Belotti et al. [38]).

### Data

The data used in this research have been primarily obtained from the Estadística de Establecimientos Sanitarios con Régimen de Internado (ESCRI), a hospital survey carried out annually by the Spanish Ministry of Health, Social Services and Equality (MSSSI) during the period 1996–2009. It provides data on capital and human resources, outcomes and accounting information related to the expenditure and investment of the units of the Spanish hospital system. The period analysed starts in 2002 (coinciding with the end of the decentralisation process of health care management to the Autonomous Communities) and ends in 2009. In order to work with a comparable sample, only public general hospitals with a minimum capacity (on average for the period 2002–2009) of 500 installed beds and allowing a longitudinal follow up during the complete period of study were included. The final sample is composed of a balanced panel of 57 Spanish public general hospitals for the period analysed (N = 456 and T = 8). Hospitals finally included in the sample are distributed geographically as follows; Andalucía (Reg01) = 13 hospitals; Aragón (Reg02) = 2; P. Asturias (Reg03) = 1; I. Baleares (Reg04) = 1; I. Canarias (Reg05) = 3; Cantabria (Reg06) = 1; Castilla-La Mancha (Reg07) = 2; Castilla y León (Reg08) = 2; Cataluña (Reg09) = 5; C. Valencia (Reg10) = 6; Extremadura (Reg11) = 2; Galicia (Reg12) = 5; Madrid (Reg13) = 7; R. Murcia (Reg14) = 1; C.F. Navarra (Reg15) = 2; País Vasco (Reg16) = 3 and La Rioja (Reg17) = 1. Additional information was also obtained from other data sources which are specified below (for each of the corresponding variables).

### Variables

#### Dependent variable

As dependent variable, the observed total costs (*TC*_*it*_) of each hospital is used. This is obtained as an aggregate of: total personnel costs (*TPEC*_*it*_);total purchase costs -other than pharmaceuticals, i.e., expenditure on diagnostic materials, sanitary and other supplies, small tools, etc.- (*TPUC*_*it*_); total pharmaceutical costs (*TPHC*_*it*_) and total capital costs (*TCAC*_*it*_). *TPEC* and *TPUC* are deflated to year 2009 prices by applying a price index calculated from the Healthcare and Other Public Administrations Gross Domestic Product, using data obtained from the Spanish National Statistics Institute (INE). *TPHC* are deflated to year 2009 prices by applying an index for Drugs, other Pharmaceutical Products and Therapeutic Supplies, obtained from INE. Finally, *TCAC* are deflated to year 2009 prices by applying an index calculated from the total Regional Gross Fixed Capital Formation, using data obtained from the Base de Datos Regionales de la Economía Española B.D.MORES.

#### Output variables

Three output variables, *y*_*mit*_, are defined by differentiating the type of hospital production. First, a variable reflecting the hospital activity in an in-patient regime (*I*_*it*_), and defined as the weighted sum of discharges in ten different hospital services is included. Hospital services provided in the ESCRI are: General Medicine; Surgery; Traumatology; Obstetrics and Gynaecologic; Paediatric; Rehabilitation; Intensive Care; Long Stay; Psychiatric and Others. To aggregate the production of the different hospital services, the weighting system used here is based on the coefficients defined by the weighted care unit Unidad Ponderada Asistencial (UPA), first developed by Bestard et al. [39]. The UPA is a unit measure of hospital production in the Spanish context prior to the development of Diagnosis Related Groups (DRG). This tool was mainly aimed at measuring the different types of hospital production by distinguishing between activities performed on an inpatient, outpatient and an emergency basis. A weighting system was then calculated upon their relative contribution to hospital costs. Second, hospital production on an outpatient basis is accounted for in our model by including a variable composed of the weighted sum of visits (first and subsequent), ambulatory surgical procedures, day-care unit visits and home hospitalisation visits (*O*_*it*_). Finally, a variable accounting for the activity in the emergency department (*E*_*it*_) and defined as the total weighted number of discharges (i.e., leading or not to in-hospital stays) at this department is included in Eq (2). In both outpatient and emergency variables, hospital production is aggregated by means of weights derived from the UPA system. Weight units for outpatient care were derived by re-scaling the weights defined by UPAs taking the first visit as the unit, so that: first visit (weight = 0.25/0.25 UPAs = 1), subsequent visit (weight = 0.15/0.25 UPAs = 0.60), ambulatory surgical procedures (weight = 1.5/0.25 UPAs = 6), day-care units (weight = 0.75/0.25 UPAs = 3) and home hospitalisation (weight = 0.75/0.25 UPAs = 3). Weight unit for emergency care was derived by re-scaling the weight defined by UPAs, taking the first visit as the unit, so that: weight = 0.5/0.25 UPAs = 2.

#### Input prices

A price for the capital input, ((*WAC*_*it*_, is defined here as the total annual depreciation of capital assets, considering all the hospital facilities as the unit of capital stock. Thus, we follow a similar approach to that proposed by Vitikainen, Linna and Street [19], who pointed out that considering a hospital’s capital expenses per bed provides a proxy of the capital input used only for care provision on an inpatient basis, and consequently used total capital expenses as a measure of the *physical* capital stock. In addition to this, a price for the labour category, (*WLA*_*it*_), defined as the total expenditure per equivalent unit of personnel, is also included in the cost frontier. Equivalent units are calculated as an aggregate of personnel holding a full-time, part-time and collaborator position. Thus, according to Ley [40], the following weights are assigned to each category: full-time (weight = 1); part-time (weight = 0.5) and collaborator (weight = 0.3). Finally, two input prices referring to the consumption of pharmaceutical inputs (*WPH*_*it*_) and materials and services (*WPU*_*it*_) are included in the cost frontier. *WPH*_*it*_ is calculated as the yearly total expenditure on drugs and other pharmaceutical products, and *WPU*_*it*_ as the yearly total expenditure on materials and services -other than pharmaceuticals. A proxy for the pharmacy input (*upasphar*) is defined as the weighted sum (following the UPA weighting system) of discharges in ten different hospital services, visits (diagnosis or review), ambulatory surgical procedures, day-care unit visits, home hospitalisation visits and discharges of the emergency department. The proxy for the materials and services input (*upasmatss*) is calculated as *upasphar* but considering the weighted sum of days of stay instead of discharges. Discharges are regarded here as a better proxy for pharmacy input as investment decisions on drugs of relatively higher prices might be taken in light of their relatively higher effectiveness. Input price *WPU*_*it*_ is used to normalise the cost frontier.

#### Heterogeneity variables

On the one hand, hospital observable cost heterogeneity is controlled for by including a set of exogenous variables in Eq (2). Thus, although a widely accepted measure of patients’ complexity is provided by Diagnosis Related Groups (DRGs), unfortunately, output measures provided in the ESCRI are not disaggregated by this classification system. Consequently, hospital complexity is accounted for by means of: first, a case severity index proposed by Roemer, Moustafa and Hopkins in 1968 [41] (*ROEMER*_*it*_), which is calculated by multiplying the average length of stay for each hospital by the rate resulting from dividing its occupancy rate by the average occupancy rate of all hospitals in the sample. The average length of stay is calculated by dividing the days of stay by the number of discharges in a year, whereas the occupancy rate is calculated by dividing the days of stay by the number of beds during the year. Roemer, Moustafa and Hopkins [41] argue that length of stay depends not only on severity but also on external factors related to supply (e.g., bed availability) and to demand pressures. Thus, adjustment is made to isolate the length of stay from the influence of these factors, so that instead it depends on patient morbidity [41]. Second, hospital complexity is also approximated through the *Rotation Index* (*ROTATION*_*it*_), which is obtained by dividing total hospital discharges by total number of beds. Hospitals facing more complex and chronic cases will be able to rotate beds less frequently.

Following a similar approach to that proposed by Vitikainen, Linna and Street [19], the specialisation level is approximated by means of concentration indexes. In particular, the degree of specialisation within the hospital inpatient activity is approximated by the share of the largest hospital service in terms of discharges (*C*1_*INP*_*it*_). The concentration index within the outpatient activity is calculated as the share of the largest hospital service in terms of visits (*C*1_*OUTP*_*it*_).

The possibility of substituting in-hospital activity by any type of outpatient care has been accounted for in the cost frontier by means of dummy variables indicating the availability of an associated dependent outpatient clinic (*DOUTP*_*CLINIC*_*it*_), following the work by García [24].

Following González et al. [26], to evaluate the impact of hospital technology on hospital costs, a variable indicating the number of different types of high-technology equipment available (*TECH*_*INDEX*_*it*_) is introduced as a cost-driver in equation (2). The following ten categories of equipment provided in the ESCRI have been considered: X-ray machines; Computerized Axial Tomography imaging-scanner; Magnetic Resonance imaging-scanner; Extracorporeal Shock Wave Lithotripsy equipment; Catheterization-Laboratory rooms; Digital Angiography equipment; Gamma cameras; Cobalt machines; Linear accelerators and Haemodialysis machines.

As the majority of units in the sample of study carry out training of healthcare personnel, differences in costs due to the degree of teaching activity performed have been controlled for by the ratio of the total number of resident physicians and midwives to the total number of doctors and nurses (*RATIO*_*RESID*_*it*_). The underlying premise is that hospitals with a relatively higher number of healthcare personnel undergoing training would present a relatively higher level of teaching activity and, consequently, are expected to present a different cost structure.

In order to account for the fact that differences in hospitals’ cost structures may arise from differences in the quality of care, the following two proxies related the healthcare personnel devoted to the provision of hospital care have been included in the Eq (2): first, the total number of nurses per doctor (*RATIO*_*NURSES*_*it*_) and second, the total number of nursing assistants per nurse (*RATIO*_*ASSIST*_*it*_).

On the other hand, hospital unobservable cost heterogeneity is accounted for by including a set of individual dummy variables *Dhosp*_*qi*_, in the cost frontier. *Dh*01_*reg*17 is taken as the reference category.

## Results

Variables used, as well as their descriptive statistics, are listed in Table 1.

**Table 1:** Definition and descriptive statistics of variables.

Variable name	Variable definition	Mean	S.D.	Min.	Max.
Cost variables	** **	** **	** **	** **	** **
Total Costs (TC)	Total hospital variable costs (in euros)	255,417,106	109,957,068	96,976,514	615,799,975
Total Personnel Costs (TPEC)	Total hospital expenditure on personnel (in euros)	163,955,730	70,225,412	67,588,745	372,434,027
Total Materials and ss Costs (TPUC)	Total hospital expenditure on purchases-other than pharmaceuticals (in euros)	60,980,504	25,742,417	20,044,125	153,751,979
Total Pharmacy Costs (TPHC)	Total hospital expenditure on pharmaceutical products (in euros)	28,035,234	17,749,146	3,055,662	126,900,000
Total Capital Costs (TCAC)	Total hospital capital depreciation (in euros)	2,445,638	2,394,473	116,678	15,400,000
Output variables					
Inpatient (I)	Weighted sum of discharges (in UPAs)	53,934	21,858	24,397	134,310
Outpatient (O)	Weighted sum of visits, ambulatory surgeries and day-hospital visits (in UPAs)	519,743	213,466	197,934	1,381,872
Emergency (E)	Weighted sum of total discharges of the emergency department (in UPAs)	311,999	116,782	104,460	722,402
Input prices					
Price of Capital (WCA)	Total hospital capital depreciation (in euros)	2,445,638	2,394,473	116,678	15,400,000
Price of Labour (WLA)	Total expenditure of personnel (in euros) per equivalent unit	44,951	8,196	30,018	94,694
Price of Pharmacy (WPH)	Total expenditure of pharmaceuticals products (in euros) per upasphar	106	52	14	446
Price of Materials and ss (WPU)	Total expenditure of materials and services (in euros) per upasmatss	109	24	55	213
Observable Heterogeneity variables					
ROEMER	Average length of stay_it_ · (Occupancy rate_it_ / Occupancy rate_t_)	7.084	1.456	3.921	12.473
ROTATION	Total hospital discharges per installed beds	44.432	6.354	28.718	73.973
C1_INPAT	Share of the largest hospital service in terms of inpatient discharges	0.358	0.066	0.192	0.575
C1_OUTPAT	Share of the largest hospital service in terms of outpatient visits	0.362	0.057	0.271	0.569
DOUTP_CLINIC	Equal to 1 if any outpatient clinic is available, 0 otherwise	0.757	0.430		
TECHNO_INDEX	Number of different types of high-technology equipment available	7.482	1.961	1.000	10.000
RATIO_RESID	Number of residents / Number of doctors and nurses	0.108	0.038	0.000	0.230
RATIO_NURSES	Number of nurses / Number of doctors	1.406	0.264	0.614	2.137
RATIO_ASSIST	Number of nurses / Number of nurses assistants	0.845	0.120	0.533	1.275

Tables 2, 3 and 4 contain the results on cost frontier estimates for the two models estimated.

**Table 2:** Cost frontier and error components estimates.

Variable	M-I^a^			M-II^b^			Variable	M-I^a^			M-II^b^		
	Coefficient		S.E.	Coefficient		S.E.		Coefficient		S.E.	Coefficient		S.E.
Constant	14.318	***	0.052	14.428	***	0.129	lwph^2^	0.132	***	0.031	0.084	***	0.024
Li	0.230	***	0.053	0.191	***	0.053	lwcali	0.070		0.044	0.031		0.041
Lo	0.208	***	0.023	0.126	***	0.025	lwcalo	-0.028		0.027	-0.009		0.024
Le	0.304	***	0.039	0.307	***	0.044	lwcale	-0.062	*	0.037	-0.028		0.036
Lwca	0.038	***	0.011	0.035	***	0.010	lwlali	-0.087		0.088	-0.052		0.071
Lwla	0.373	***	0.021	0.438	***	0.018	lwlalo	0.083		0.073	0.039		0.057
Lwph	0.189	***	0.014	0.143	***	0.012	lwlale	-0.009		0.081	0.006		0.064
li^2^	-0.478	**	0.216	-0.214		0.194	lwphli	0.233	***	0.070	0.215	***	0.058
Lilo	-0.033		0.098	-0.062		0.104	lwphlo	-0.011		0.051	0.002		0.042
Lile	0.441	***	0.151	0.314	**	0.150	lwphle	-0.223	***	0.057	-0.236	***	0.046
lo^2^	0.326	**	0.104	0.149	*	0.088	lt	-0.016		0.015	-0.009		0.010
Lole	-0.042		0.088	-0.104		0.091	lt^2^	-0.017		0.015	-0.040	***	0.013
le^2^	-0.382	**	0.188	-0.297		0.192	ltli	-0.094	***	0.029	-0.045	**	0.023
ltcac^2^	0.010		0.016	0.000		0.014	ltlo	0.031		0.022	0.022		0.018
Lwcalwla	0.071	***	0.026	0.067	***	0.022	ltle	0.032		0.027	0.011		0.023
Lwcalwph	-0.030		0.019	-0.034	**	0.017	ltlwca	0.014		0.009	0.015	**	0.007
lwla^2^	-0.218	**	0.090	-0.199	***	0.069	ltlwla	0.051	**	0.024	0.044	**	0.020
Lwlalwph	-0.080	*	0.045	-0.045		0.035	ltlwph	-0.002		0.014	0.013		0.012

^a^M-I = Model omitting heterogeneity factors

^b^M-II = Model accounting for heterogeneity factors

*significant at 10%

** significant at 5%

*** significant at 1%

**Table 3:** Cost frontier and error components estimates.

Variable	M-I^a^			M-II^b^			Variable	M-I^a^			M-II^b^		
	Coefficient		S.E.	Coefficient		S.E.		Coefficient		S.E.	Coefficient		S.E.
ROEMER				0.028	***	0.004	DH10_REG01				0.044		0.038
ROTATION				-0.002	**	0.001	DH11_REG01				0.050		0.041
C1_INPAT				-0.145		0.100	DH12_REG01				-0.197	***	0.039
C1_OUTPAT				0.061		0.095	DH13_REG01				-0.143	***	0.039
DOUTP_CLINIC				0.028		0.026	DH01_REG02				0.105	**	0.042
TECHNO_INDEX				0.000		0.003	DH02_REG02				0.283	***	0.063
RATIO_RESID				-1.047	***	0.152	DH01_REG03				0.329	***	0.060
RATIO_NURSES				-0.023		0.024	DH01_REG04				0.165	***	0.044
RATIO_ASSIST				0.146	***	0.051	DH01_REG05				0.081	*	0.047
DH01_REG01				-0.042		0.038	DH02_REG05				0.130	***	0.045
DH02_REG01				0.233	***	0.063	DH03_REG05				0.059		0.057
DH03_REG01				-0.027		0.046	DH01_REG06				0.138	**	0.054
DH04_REG01				-0.050		0.048	DH01_REG07				0.035		0.037
DH05_REG01				0.062		0.047	DH02_REG07				0.043		0.047
DH06_REG01				0.281	***	0.066	DH01_REG08				-0.080	***	0.031
DH07_REG01				0.170	**	0.070	DH02_REG08				0.024		0.033
DH08_REG01				0.456	***	0.090	DH01_REG09				0.175	**	0.069
DH09_REG01				0.181	**	0.084	DH02_REG09				-0.070		0.050

^a^M-I = Model omitting heterogeneity factors

^b^M-II = Model accounting for heterogeneity factors

*significant at 10%

** significant at 5%

*** significant at 1%

**Table 4:** Cost frontier and error components estimates.

Variable	M-I^a^			M-II^b^			Variable	M-I^a^			M-II^b^		
	Coefficient		S.E.	Coefficient		S.E.		Coefficient		S.E.	Coefficient		S.E.
DH03_REG09				0.176	**	0.080	DH03_REG13				-0.076	*	0.040
DH04_REG09				-0.260	***	0.072	DH04_REG13				0.350	***	0.066
DH05_REG09				0.380	***	0.077	DH05_REG13				0.312	***	0.062
DH01_REG10				0.019		0.040	DH06_REG13				0.447	***	0.094
DH02_REG10				-0.036		0.033	DH07_REG13				0.320	***	0.093
DH03_REG10				0.137	***	0.045	DH01_REG14				0.121	**	0.053
DH04_REG10				-0.101	**	0.049	DH01_REG15				-0.003		0.067
DH05_REG10				0.085	**	0.045	DH02_REG15				-0.040		0.049
DH06_REG10				0.495	***	0.073	DH01_REG16				-0.089		0.059
DH01_REG11				-0.106	***	0.038	DH02_REG16				0.086		0.063
DH02_REG11				0.097	**	0.043	DH03_REG16				0.090		0.064
DH01_REG12				-0.063	*	0.032							
DH02_REG12				0.032		0.040	μ	0.272	***	0.071	-0.055		0.225
DH03_REG12				0.174	***	0.060	η	-0.010		0.012	-0.427	***	0.062
DH04_REG12				0.039		0.069	γ = (σ_u_^2^ + σ_v_^2^) / σ^2^	0.910	***	0.037	0.936	**	0.074
DH05_REG12				0.009		0.031							
DH01_REG13				-0.131	**	0.051	Log-likelihood	667.467			896.689		
DH02_REG13				-0.097	**	0.043							

^a^M-I = Model omitting heterogeneity factors

^b^M-II = Model accounting for heterogeneity factors

*significant at 10%

** significant at 5%

*** significant at 1%

Cost frontiers specified satisfy the properties of non-negativity and homogeneity of degree 1 in input prices. Fulfilment of this property was imposed prior to the estimation process by the normalisation of both costs and input prices. Concerning the property of non-decreasing in input prices, it is verified that this property is satisfied by at least 96% of observations in all models estimated. Property of non-decreasing in outputs is satisfied in a minimum of 97% of observations in all specifications. Finally, to check whether the functions satisfy the property of concavity in input prices, it was evaluated whether the matrix of derivatives of second order with respect to input prices was negative semi-definite. However, the sequence of signs of principal minor was not as desired. This could be explained by the positive sign of the partial derivative of second order corresponding to the pharmacy input, which may indicate the inelastic nature of the demand for this factor during the period analysed.

The proportion of the variance of the total error that is due to variance in the inefficiency component, calculated as $\gamma =\sigma_{u}^{2}/(\sigma_{u}^{2}+\sigma_{\nu }^{2})$, dominates due to the variance of the random noise component. Thus, the former accounts for 91% and 93.6% of cost variability in Models I and II, respectively, which confirms the presence of inefficiency in the hospital activity analysed. Moreover, since parameter η is negative and significant in Model II, cost efficiency decreased during the period of study. However, time did not have a significant impact on cost efficiency variation under Model I. Regarding parameter μ, it was not statistically significant in Model II, thus, the half-normal distribution cannot be rejected and model collapses to that proposed by Aigner, Lovell and Schmidt [42], though assuming efficiency to be time-varying (for more details, see [43]).

In order to guide the model selection, the appropriateness of a Cobb-Douglas functional form is evaluated as an alternative to the translog model specified. This hypothesis is tested by imposing null values to squared terms and cross-products (i.e., H_0_: *β*_*mj*_ = *β*_*nk*_ = *β*_*mn*_ = *β*_*tt*_ = *β*_*mt*_ = *β*_*nt*_ = 0). The Cobb-Douglas form is rejected since λ = 126.76 (p-value<0.05), so that the flexible translog is the preferred functional form. Our model is further validated by testing the joint significance of the heterogeneity variables. Thus, a general likelihood (LR) test is implemented to evaluate the hypothesis H_0_: *θ*_1_ = … = *θ*_9_ = *ρ*_1_ = … = *ρ*_56_ = 0. This hypothesis is rejected (λ = 458.44 and p-value < 0.01), which indicates that heterogeneity variables included, both *h*_*pit*_ and *Dhosp*_*qi*_, have, as a group, a significant impact on costs. Thus, these results corroborate the better fit of Model II to the data analysed when compared to the alternative specification.

Results for the TFP index change decomposition for models estimated are reported in Table 5.

**Table 5:** TFP index change decomposition.

		2002/09			
		TFP-I^a^	TFP-E^b^	TFP-T^c^	TFP-S^d^
**Model-I**^**e**^	**Mean**	-0.028	-0.419	0.021	0.371
	**S.D**	0.848	0.278	0.950	0.530
**Model-II**^**f**^	**Mean**	-1.330	-1.007	-0.032	-0.291
	**S.D**	6.946	0.776	0.481	6.871
**V(II-I)**^**g**^	**Mean**	-1.303	-0.588	-0.053	-0.661

^a^TFP-I = Total Factor Productivity Index Change

^b^TFP-E = Cost Efficiency Change

^c^TFP-T = Technical Change

^d^TFP-S = Scale Economies Effect

^e^Model-I = Model omitting heterogeneity factors

^f^Model-II = Model accounting for heterogeneity factors

^g^V(II-I) = TFP (Model II)—TFP (Model I)

Comparison of results on the TFP index change between models provides a measure of the biased caused in hospital productivity by not controlling for cost heterogeneity. Thus, when these elements are omitted (i.e., Model I), hospital productivity remains invariant during the period analysed. However, an overall decrease in mean hospital productivity levels is obtained when heterogeneity is accounted for (i.e., Model II). In particular, findings show a difference between a productivity decrease at an annual average rate of -0.028% (Model I) and a productivity decrease at an annual average rate of -1.330% (Model II) when heterogeneity is controlled for (i.e., a bias of 1.303 percentage points in the overall TFP-I for not controlling for heterogeneity). Further analysis also reveals differences in the sources of variation. When heterogeneity factors are considered (i.e., Model II), decreases in hospital productivity are mainly explained by a decrease in the cost efficiency change component. Moreover, results point to the negative contribution of scale economies effects, whereas a positive contribution of this component is obtained when heterogeneity is omitted. In the latter case, scale economies effect contributes to ameliorate decreases in the overall TFP index. Concerning the technical change component, its contribution resulted practically negligible under both specifications. In addition to this, the component that measures variations in the TFP-I due to possible bias introduced by the aggregation of inputs is insignificant. Moreover, the lack of availability of prices of hospital services prevents us from measuring the variation in productivity due to bias introduced by the aggregation of outputs.

Table 6 contains results on the sample mean cost efficiency indexes for the three models estimated.

**Table 6:** Cost efficiency index.

	Model-I^a^		Model-II^b^		V(II-I)^c^
	Mean	S.D.	Mean	S.D.	Mean
**Cost efficiency index**	0.730	0.124	0.974	0.035	0.244

^a^Model-I = Cost efficiency index under model omitting heterogeneity factors

^b^Model-II = Cost efficiency index under model accounting for heterogeneity factors

^c^V(II-I) = Cost efficiency index (Model II)—Cost efficiency index (Model I)

A measure of the bias caused when not controlling for cost heterogeneity can be obtained comparing the cost efficiency indexes estimates of the alternative models. Thus, the comparison of efficiency indexes yielded by Models I and II reveals an increase in cost efficiency from 0.730 (Model I) to 0.974 (Model II), so that the omission of heterogeneity leads to an overall decrease of 24.4 percentage points.

The estimated coefficients of the variables included to control for heterogeneity in Model II also provide interesting information on the hospital technology of production. Thus, more complex hospitals present relatively higher costs, as indicated by the significant positive and negative coefficients of variables *ROEMER* and *ROTATION*, respectively. Moreover, hospitals with a higher teaching activity have relatively lower costs, as the variable *RATIO_RESID* resulted significant and negative. Furthermore, hospitals with a higher number of nursing assistants per nurse present relatively higher costs, as evidenced by the significant positive coefficient of variable *RATIO_ASSIST*. Finally, results on individual dummy variables reveal the existence of unobserved hospital characteristics leading to significant differences in costs.

## Discussion

This study has used a stochastic cost frontier approach to quantify the bias that not accounting for heterogeneity in costs may cause in hospital productivity measurements in the Spanish public hospital sector during the period 2002–2009. Thus, findings indicate that overall productivity remained practically invariant during the period analysed when heterogeneity is omitted. However, if heterogeneity is controlled for, an annual average rate of -1.330% in hospital productivity is obtained. This leads to an estimated bias in the annual average variation rate of 1.303 percentage points, attributable to cost heterogeneity omission. Moreover, contribution of each component in productivity levels also varies between models. Hence, when heterogeneity is accounted for, decreases in TFP index are mainly driven by a decrease in cost efficiency levels. In addition to this, scale economies effects also contribute to reductions in productivity. However, when heterogeneity elements are omitted, scale economies effects contribute to improve hospital productivity. Contribution of the technical change component is negligible in all scenarios. Hence, the correct identification of the contribution of each component to the productivity change is crucial to the evaluation of the productive behaviour of hospitals and to the consequent design of appropriate policies. For example, in a scenario where heterogeneity is not controlled for, inefficient behaviours might be offset by improving the promotion of a better utilisation of the scale of operations. However, cost efficiency change turns out to be relatively more relevant to productivity change when accounting for cost heterogeneity. Therefore, the premise of Greene [11] for efficiency measurement can be also extended to productivity measurement. This constitutes the main contribution of this paper, as we are not aware of any previous study analysing the effects of not controlling for cost heterogeneity on hospital productivity levels.

Decreases in efficiency levels obtained may be motivated by the increasing resources devoted to the provision of healthcare in Spain during the period 2002–2009. The public health expenditure in Spain grew at an annual average rate of 9% during the period 2002–2009 (data obtained from the MSSSI), compared with 5% Gross Domestic Product growth rate (data obtained from the INE). Moreover, the decentralisation process of the Spanish National Health System was completed in 2002, which gave rise to regional autonomy in the decision-making for the resource allocation. Consequently, the period analysed was characterised by managerial decisions leading to the acquisition of new high-technology equipment or to the construction and/or to the expansion of facilities. This greater availability of resources could have discouraged efficient behaviours and cost containment, as identified elsewhere by Rumbold et al. [44]. Moreover, organisational changes that may have been introduced may not have had a direct impact on performance levels, requiring in some cases an adaptation period by health care providers.

A measure of the bias caused in cost efficiency indexes is also obtained in this research. If heterogeneity factors are not controlled for in the cost frontier when modelling the hospital productive technology, the mean cost efficiency index during the period analysis is 0.730. When accounting for cost heterogeneity factors, the mean increases up to 0.974. Hence, the omission of heterogeneity leads to a bias of 24.4 percentage points in the mean cost efficiency. Furthermore, these results are in line with those reported by Widmer, Zweifel and Farsi [30] and Widmer [31], who also reported bias in hospital cost efficiency indexes in Switzerland when omitting sources of hospital cost heterogeneity. Thus, not accounting for hospital cost heterogeneity might give rise to managerial decisions based on miscalculated overruns. For instance, under the omission of cost heterogeneity, an average reduction of annual hospital costs of about 77.6 million € would be obtained by improving hospital cost efficiency; however, this amount would be reduced to 7.2 million if we adjust for heterogeneity sources.

The analysis of the estimated coefficients of the variables introduced to adjust for heterogeneity in Model II reveals that direction of results obtained are as expected. Thus, higher cost levels for those relatively more complex hospitals in Spain were also obtained by Quintana [13], González and Barber [14] and Wagstaff and López [15]. Moreover, hospitals with a higher number of residents per healthcare personnel present relatively lower costs. However, given that our proxy is defined as the number of resident physicians and midwives under training at hospitals to the number of doctors and nurses, findings may be explained by the relatively lower salaries of residents. Moreover, hospitals devoting more nursing assistants to the provision of care have relatively higher costs, confirming the trade-off between quality of care and efficiency.

Therefore, these findings highlight the need to account for the influence of heterogeneity factors in the measurement of hospital performance to prevent decision-makers from adopting inadequate policies on resource allocation.

This research provides evidence on the bias that omissions of hospital cost heterogeneity variables may cause in productivity measures. In this regard, substantial differences were found between results arising from the models used for performance measurement. First, while overall productivity practically remains invariant when sources of heterogeneity are not accounted for, a decrease in productivity is obtained when these elements are controlled for. Differentiated results are also encountered when analysing the contribution of each component of the TFP index. Thus, when adjusting for heterogeneity factors, the scale economies effects reduce productivity. However, if omitting hospital cost heterogeneity, economies of scale effects contribute to ameliorate decreases in productivity. The contribution of the technical change component to productivity resulted negligible in both models estimated. In addition to this, the higher inefficiency index obtained here for a restricted model including merely inputs and outputs, compared to that obtained under its extended version which includes cost heterogeneity variables, provides evidence of the bias introduced in cost efficiency indexes.

## Supporting information

S1 File Dataset used for analysis. (XLSX)
